# Effect of Time to Start of Biologic Therapy on Treatment Response in Childhood Arthritis: Results From the UCAN CAN‐DU Cohort

**DOI:** 10.1002/art.43401

**Published:** 2026-01-16

**Authors:** Jelleke B. de Jonge, Sytze de Roock, Dieneke Schonenberg‐Meinema, J. Merlijn van den Berg, Deborah A. Marshall, Sebastiaan J. Vastert, Rae S. M. Yeung, Joost F. Swart, Susanne M. Benseler, the UCAN CAN‐DU and UCAN CURE consortia, Adam Huber, Bianca Lang, Chelsea DeCoste, Elizabeth Stringer, Suzanne Ramsey, Alan Rosenberg, Kate Neufeld, Mehul Jariwala, Tristan Kerr, Alexander Mosoiu, Alisa Rachlis, Amy Xu, Arthur Cheng, Brenleigh Jebb, Brian Feldman, Bruno Pereira, Deborah Levy, Dilan Dissanayake, Elizaveta Limenis, Evelyn Rozenblyum, Harper Cheng, Jennifer Ji Young Lee, Lynn Spiegel, Rayfel Schneider, Ronald Laxer, Ruud Verstegen, Shirley Tse, Trang Duong, Andrea Human, David Cabral, Herman Tam, Jaime Guzman, Kim Morishita, Kristin Houghton, Lori Tucker, Mercedes Chan, Ross Petty, Tommy Gerschman, Annet van Royen‐Kerkhof, Berent Prakken, Erika Van Nieuwenhove, Marc Jansen, Nico Wulffraat, Ciarán Duffy, Nadia Luca, Roman Jurencak, Tala El Tal, Claire LeBlanc, Gaëlle Chédeville, Piya Lahiry, Rosie Scuccimarri, Sarah Campillo, Clare Hutchinson, Daniah Basodan, Dax G. Rumsey, Hon Yan Ng, Jeanine McColl, Lillian Lim, Tara McGrath, Danielle Brinkman, Petra Hissink Muller, Elizabeth Legger, Wineke Armbrust, Ellen Schatorje, Esther Hoppenreijs, Elodie Boudes, Gillian Currie, Heinrike Schmeling, Muhammed Dhalla, Nicole Johnson, Paivi Miettunen, Ravneet Sran, Rebeka Stevenson, Erkan Demirkaya, Jonathan Park, Roberta Berard, Giske Biesbroek, Mariken Gruppen, Gordon Soon, Joseph Cafazzo, Liane Heale, Michelle Batthish, Tania Cellucci, Lily Lim, Maarten IJzerman, Marinka Twilt, Marleen Verkaaik, Philomine van Pelt, Sylvia Kamphuis, Michelle Kip, Nickolas Blanchette, Paul Dancey, Regina de Geus

**Affiliations:** ^1^ Division of Pediatrics, Department of Pediatric Rheumatology Wilhelmina Children's Hospital, University Medical Center Utrecht the Netherlands; ^2^ Faculty of Medicine Utrecht University the Netherlands; ^3^ Center for Translational Immunology, University Medical Center Utrecht, Utrecht University the Netherlands; ^4^ Department of Pediatric Immunology, Rheumatology and Infectious Diseases Emma Children's Hospital, Amsterdam University Medical Centers, University of Amsterdam the Netherlands; ^5^ Departments of Community Health Services and Medicine McCaig Institute for Bone and Joint Health, O'Brien Institute for Public Health, Cumming School of Medicine, University of Calgary Calgary Alberta Canada; ^6^ Alberta Children's Hospital Research Institute, Cumming School of Medicine, University of Calgary Calgary Alberta Canada; ^7^ Division of Rheumatology, Department of Paediatrics Immunology and Institute of Medical Science, The Hospital for Sick Children, University of Toronto Toronto Ontario Canada; ^8^ Division of Rheumatology, Department of Pediatrics Alberta Children's Hospital, Cumming School of Medicine, University of Calgary Calgary Alberta Canada; ^9^ Rheumatology, Paediatrics Children's Health Ireland Dublin Ireland

## Abstract

**Objective:**

To estimate the effect of time from symptom onset to start of biologic treatment on achieving inactive arthritis within six months in a cohort of patients with juvenile idiopathic arthritis (JIA).

**Methods:**

The international UCAN CAN‐DU study prospectively enrolled patients with JIA across Canada and the Netherlands. A nested cohort study was performed and biologic‐naive patients with nonsystemic JIA were included at the start of biologic therapy. The primary outcome was inactive arthritis at six months. Demographics, disease‐related parameters, and treatment response were compared using (non)parametric tests among early (time symptom onset to biologic start: 0–6 months), intermediate (7–12 months), and late (13–24 months) treatment groups. A logistic regression model analyzed the effect of time to biologic start on the response at six months, adjusting for active joint count and physician global assessment. A graphical representation of the model was created.

**Results:**

One hundred and thirty children with JIA were included (early: n = 35; intermediate: n = 46; late: n = 49), 66% were female, and the median age at symptom onset was 11.0 years. The proportion of patients that reach inactive arthritis in the early starters (83%) was significantly higher than in late starters (57%). For each month of delay to the start of biologic treatment, the adjusted odds of having active arthritis after six months of therapy was 1.09 (interquartile range: 1.02–1.17, *P* = 0.009).

**Conclusion:**

Early start of biologic therapies in patients with JIA was associated with a higher proportion of patients reaching inactive arthritis within six months, suggesting a window of opportunity to control disease activity.

## INTRODUCTION

Juvenile idiopathic arthritis (JIA) is the most common chronic rheumatic disease in children and a significant cause of short‐ and long‐term disability.^1^ For the majority of patients, JIA will persist into adulthood, leading to a diminished quality of life.^2^ By definition, arthritis needs to persist for more than six weeks before the diagnosis can be made.^3^ The diagnostic delay is usually much longer with a median interval of 20 weeks from symptom onset to first assessment by a pediatric rheumatology team.^4^ Another barrier to fast effective therapy are the current JIA treatment guidelines that are anchored in a “step‐up approach.”^5^

JIA therapy commonly follows the sequence of nonsteroidal anti‐inflammatory drugs, intra‐articular steroid injections, conventional synthetic disease‐modifying antirheumatic drugs (csDMARDs), and, as a final step, biologic therapies. Since their introduction more than 25 years ago, the dogma prevailed that biologic treatment is to be started only after the patient failed to respond to csDMARD, an approach mainly driven by a difference in drug costs. Methotrexate (MTX) is the most frequently used csDMARD and controls arthritis and induces inactive disease in only around 30% of patients with JIA at two years.^6^ More importantly, MTX is poorly tolerated. Up to 50% of patients receiving MTX mention nausea and discomfort,^7^ amplifying the suffering of children living with arthritis.

This mandatory step‐up approach causes a long trial‐and‐error period between the diagnosis of JIA and the start of biologics, which is the most effective therapy for the majority of patients with JIA. Exposure to biologic agents is relatively uncommon in the first year of follow‐up; however, a consistent increase is found at both the three‐ and five‐year time points in both cumulative exposure and point prevalence use, such that at five years, 42% of the cohort had been exposed to a biologic.^8^ Previous studies have focused on the start time of biologic therapy by comparing a cohort of patients following different treatment strategies, such as the step‐up approach and immediate biologic start.^9^, ^10^, ^11^ These studies showed that an early start of biologic therapy after diagnosis can alter the disease trajectory compared to patients with JIA starting on csDMARDs.^9^, ^10^, ^11^ From a biologic perspective, the time between inflammation with subsequent symptom onset and the initiation of biologic therapy might be more relevant than the time between diagnosis and biologic therapy.

There are convincing data that there is a window of opportunity in the systemic form of JIA, in which an early start of a biologic leads to successful discontinuation of therapy in the majority of patients.^12^ The hypothesis that there is a limited period since onset in which biologic therapy can be most successful has been postulated for nonsystemic JIA as well.^13^ This hypothesis is that the inflammation increases progressively in a step‐wise manner, reflecting the accumulation of joints that have acquired durable changes, placing them at a higher risk of subsequent flare or persistent active disease. Effective treatment should arrest this step‐wise escalation of risk, providing a rolling window of opportunity to prevent further joint accumulation. Proof for this hypothesis has not yet been delivered but is only anecdotal.

Optimal timing of effective treatment is beneficial for clinical outcomes, patients’ quality of life, and patient and societal burden in the short‐ and long‐term. Therefore, the hypothesis that a longer time between symptom onset and initiation of biologic therapy negatively affects the probability of reaching inactive arthritis is formally tested here in an international, prospectively observed cohort of patients with JIA. The aims of the study were (1) to describe a cohort of patients with nonsystemic JIA starting with biologic treatment, (2) to compare the proportion of patients that reach inactive arthritis in early, intermediate, and late biologic treatment groups, and (3) to analyze the impact of time from symptom onset to start of biologic treatment on early outcome.

## METHODS

The international multicenter UCAN CAN‐DU study prospectively enrolled children with arthritis across Canada and the Netherlands (see Appendix A for a list of UCAN CAN‐DU and UCAN CURE consortia members). All patients participating in the UCAN CAN‐DU study provided informed consent to participate in the study. Between April 2019 and November 2024, a nested cohort study was performed within the UCAN CAN‐DU cohort, which included patients from eight centers in Canada and six centers in the Netherlands. Patients with JIA were included in this nested study if they (1) started biologic treatment for arthritis (active joint count [AJC] > 0) within 24 months after symptom onset, (2) were biologic‐naive at the start of biologic therapy, and (3) had a six‐month follow‐up visit after the start of biologic treatment (six‐month follow‐up visits are defined as a follow‐up visit within four to eight months by the UCAN CAN‐DU study protocol). Patients with systemic JIA (sJIA) were excluded from this study. Patients with a six‐month follow‐up visit were compared to those not included in the study because their follow‐up visit was outside of the defined timeframe. Patients were comparable regarding the disease status at the start of biologic therapy (Supplementary Table 1). Physician‐ and patient/family‐derived data were prospectively captured in the designated UCAN eHealth platform. The study was approved by the Institutional Research Ethical Board of the University Medical Center Utrecht (no. 18‐474) for the Netherlands and by the Conjoint Health Research Ethics Board, University of Calgary for Canada (REB17‐1563).

### Demographic, clinical data, and global measures

Demographic characteristics included age at symptom onset, biologic sex, and country of residence. International League of Associations for Rheumatology JIA subtypes were reported by the treating physicians as diagnostic criteria. In the analysis, extended oligoarticular JIA and polyarticular JIA were collectively referred to as polyarticular course JIA, whereas (persistent) oligoarticular JIA were referred to as oligoarticular course JIA. Clinical variables included JIA‐related articular and extra‐articular manifestations, including the number and distribution of affected joints and enthesitis and overall AJC. Active joints were assessed in 83 joints (carpometacarpal I joints [2×], distal interphalangeal joints in the foot [8×], and sacroiliac joints [2×]) in addition to the clinical Juvenile Arthritis Disease Activity Score (cJADAS) 71 joints,^14^ and a joint was considered active if it was swollen or had both signs of tenderness and limited range of motion. In case of doubt (limitation and/or pain), imaging was performed on the joints under supervision of the treating physician. All global measures were captured on digital visual analog scales ranging from 0 to 10 with 0.5‐point increments, including Physician Global Assessment (PhGA) of disease activity and Patient/Parent Global Assessment (PPGA). cJADAS10 scores were calculated based on the AJC, PhGA, and PPGA. Variables were collected, and assessments were performed at all visits.

### Biologic therapy and stratification

Treatment data included the medication type, dose and route, and start and stop date of all therapies, including biologic and conventional JIA treatments. Time to biologic treatment was defined as time from symptom onset to the start date of the first biologic treatment. Based on this, patients were stratified into three different treatment groups: (1) early treatment group, in which the time from symptom onset to biologic therapy was 0 to 6 months, (2) intermediate treatment group (7–12 months), and (3) late treatment group (13–24 months) (Supplementary Figure 1).

### Outcome

The outcome of biologic therapy was inactive arthritis defined as no evidence of active joints (AJC = 0) at six months after the start of biologic treatment. An AJC of zero was chosen as an outcome measurement, as this frequently guides decisions around effectiveness in real life. To account for extra‐articular manifestations, an AJC + PhGA score of <1 was used as a secondary outcome measurement.

### Analysis

Descriptive statistics summarize the patient characteristics. Comparisons were made using parametric and nonparametric tests as appropriate, including Pearson's chi‐square test, Fisher's exact test, and Kruskal–Wallis rank sum test. To test whether patients were comparable when treatment was initiated during their first hospital visit, comparisons were made using clinical data from available diagnosis visits in which patients were still treatment‐naive. Moreover, to show that patients had a comparable chance of starting a biologic early or later, the baseline characteristics age at symptom onset, AJC, and PhGA were modeled to predict the time to biologic treatment start, and the predicted and observed time to biologic treatment start were compared. Next, a logistic regression model was constructed to assess the effect of time between symptom onset and biologic start on the primary outcome, adjusted for other variables. Variables, measured at the start of biologic therapy, with a statistically relevant (*P* < 0.05) effect on the primary outcome were included in the model, as well as variables with clinical relevance (prior use of csDMARD). A potential interaction between AJC and PhGA was examined to account for their possible relationship given that PhGA is partially dependent on AJC but also on the presence of enthesitis, uveitis, and other patient characteristics. cJADAS10 was not included in the model because this composite score simply adds the information from the variables PhGA, PPGA, and AJC that are part of the univariate analysis. In addition, differences across centers were not accounted for in the models, as these partially underlie differences in treatment. A continuous variable for time between symptom onset and biologic start was used. To identify the optimal model, a backward step‐wise selection of variables was performed based on Akaike's information criterion and variance inflation factors were calculated to check for collinearity in the model. Models were compared using the likelihood ratio test. All analyses were performed in R version 4.4.1 software^15^ using the package tidyverse (version 2.0.0)^16^ and lme4 (version 1.1.35.3).^17^

## RESULTS

A total of 130 children was included, 86 were female patients (66%), and the median age at symptom onset was 11.0 years (interquartile range [IQR]: 3.8–13.4). Of these patients, 35 (26.9%) were in the early treatment group, 46 (35.4%) in the intermediate treatment group, and 49 (37.7%) in the late treatment group. Overall, 98 (75%) of the patients were treated in Dutch centers, with a significantly higher proportion of the patients treated in Dutch centers in the early treatment group (94%) compared to the intermediate (72%) and late (65%) treatment groups. A total of 83 children (64%) used csDMARDs before the start of biologic therapy, with a significantly lower proportion of patients using csDMARDs in the early treatment group (37%) compared to the intermediate (78%) and late (69%) treatment groups (Pearson's chi‐square test, *P* < 0.001). The use of csDMARDs during biologic treatment was comparable between treatment groups. The predominantly targeted biologic pathway was tumor necrosis factor α (97%) across all treatment groups; other targeted biologic pathways were interleukin‐6 (2%, tocilizumab) and JAK (1%, tofacitinib). Moreover, age at symptom onset, percentage of female patients, AJC, global assessment of both physicians and patients/parents, cJADAS10, and JIA subtype were consistent across the groups (Table 1). Complete data were available in all 130 patients for all variables, except for PPGA and cJADAS10, for which 68% was complete.

**Table 1:** Characteristics of children with JIA at the start of biologic treatment stratified by the timing of treatment since symptom onset in three treatment groups*

		Start of biologic treatment			
Characteristic	Overall n = 130	Early (0–6 months) n = 35	Intermediate (7–12 months) n = 46	Late (13–24 months) n = 49	*P* value
Age at onset	11.0 (3.8, 13.4)	10.2 (3.5, 14.2)	10.1 (3.5, 12.9)	11.4 (5.3, 13.5)	0.5^c^
Sex (female)	86 (66)	24 (69)	33 (72)	29 (59)	0.4^d^
Country of residence (the Netherlands)	98 (75)	33 (94)	33 (72)	32 (65)	0.008^a^ ^,^ ^d^
AJC	4.0 (2.0, 7.0)	5.0 (2.0, 10.5)	4.5 (3.0, 8.0)	3.0 (2.0, 6.0)	0.12^c^
PhGA	3.4 (2.0, 5.0)	4.5 (2.0, 6.0)	3.0 (2.0, 4.9)	3.5 (2.5, 4.5)	0.3^c^
PPGA^b^	4.8 (2.4, 6.8)	5.5 (2.1, 7.0)	5.5 (3.2, 7.0)	4.0 (2.2, 6.0)	0.3^c^
cJADAS10^b^	12.5 (8.9, 17.0)	15.1 (9.5, 18.0)	14.0 (10.8, 18.0)	11.0 (7.3, 14.3)	0.058^c^
Months to biologic treatment	9.0 (5.9, 15.4)	4.0 (2.8, 5.1)	8.6 (7.3, 9.5)	16.8 (14.7, 19.0)	<0.001^a^ ^,^ ^c^
csDMARDs use before biologics	83 (64)	13 (37)	36 (78)	34 (69)	<0.001^a^ ^,^ ^d^
csDMARDs continued or started	95 (73)	24 (69)	36 (78)	35 (71)	0.6^d^
Biologic therapy					>0.9^e^
TNFα inhibition	126 (97)	34 (97)	44 (96)	48 (98)	
IL‐6 inhibition	3 (2.3)	1 (2.9)	1 (2.2)	1 (2.0)	
JAK inhibition	1 (0.8)	0 (0)	1 (2.2)	0 (0)	
JIA subtype					0.6^e^
Enthesitis arthritis	31 (24)	6 (17)	11 (24)	14 (29)	
Oligoarticular course JIA	43 (33)	14 (40)	11 (24)	18 (37)	
Polyarticular course JIA	50 (38)	13 (37)	21 (46)	16 (33)	
Psoriatic arthritis	3 (2)	1 (3)	2 (4)	0 (0)	
Undifferentiated JIA	3 (2)	1 (3)	1 (2)	1 (2)	

* Values presented as median (interquartile range) or n (%). AJC, Active Joint Count; cJADAS10, clinical Juvenile Arthritis Disease Activity Score 10 joints; csDMARD, conventional synthetic Disease‐Modifying Antirheumatic Drug; IL‐6, Interleukin‐6; JIA, Juvenile Idiopathic Arthritis; PhGA, Physician Global Assessment; PPGA, Patient/Parent Global Assessment; TNFα, Tumor Necrosis Factor α.

^a^ Statistically significant at *P* < 0.05.

^b^ Data complete for 68% of the patients.

^c^ Kruskal‐Wallis rank sum test.

^d^ Pearon's chi‐square test.

^e^ Fisher's exact test.

### Sensitivity analysis at diagnosis visit

A total of 91 of the 130 patients had an available diagnosis visit on which a subanalysis was performed to check whether the early, intermediate, and late treatment groups were comparable during the visits in which treatment was initiated. This analysis showed that at diagnosis visits, in which all patients were still treatment‐naive for csDMARDs and biologics, the variables AJC, PhGA, PPGA, and JIA subtype were all comparable across the three treatment groups (Table 2). In addition, only weak correlation was found between the predicted “months to biologic start” (based on a linear model including the variables age at symptom onset, AJC, and PhGA at first hospital visit) and the observed “months to biologic start,” indicating that the variables of treatment‐naive patients could not predict when a biologic was initiated (Supplementary Figure 2).

**Table 2:** Comparison of JIA patient‐ and disease‐related factors at the treatment‐naive diagnosis visit, stratified by timing of treatment since symptom onset in three treatment groups*

		Start of biologic treatment			
Characteristic at diagnosis visit	Overall n = 91	Early (0–6 months) n = 27	Intermediate (7–12 months) n = 28	Late (13–24 months) n = 36	*P* value
Age at onset	11.4 (4.9, 13.4)	10.2 (3.6, 13.9)	10.9 (4.3, 12.9)	11.6 (9.0, 13.5)	0.7^d^
Sex (female)	56 (62)	18 (67)	17 (61)	21 (58)	0.8^e^
Country of residence (the Netherlands)	72 (79)	26 (96)	24 (86)	22 (61)	0.002^a^ ^,^ ^e^
AJC	4.0 (2.0, 7.5)	4.0 (2.0, 11.0)	4.5 (2.0, 7.0)	2.5 (1.8, 4.3)	0.4^d^
PhGA	4.0 (3.0, 6.0)	3.5 (2.6, 6.8)	4.0 (2.8, 5.0)	3.8 (3.0, 5.3)	>0.9^d^
PPGA^b^	5.0 (3.6, 7.0)	4.5 (1.8, 6.8)	5.5 (4.8, 6.7)	4.6 (3.9, 6.7)	0.6^d^
cJADAS10^b^	13.5 (9.4, 17.3)	15.0 (9.6, 17.5)	13.6 (11.0, 17.2)	11.5 (8.5, 16.8)	0.7^d^
JIA subtype^c^					0.4^f^
Enthesitis arthritis	20 (25)	3 (14)	7 (28)	10 (29)	
Oligoarticular course JIA	29 (36)	6 (27)	8 (32)	15 (44)	
Polyarticular course JIA	27 (33)	11 (50)	8 (32)	8 (24)	
Psoriatic arthritis	2 (2)	1 (5)	1 (4)	0 (0)	
Undifferentiated JIA	3 (4)	1 (5)	1 (4)	1 (3)	

* Patient characteristics were measured at the diagnosis visit (first available hospital visit, treatment‐naive for csDMARDs and biologics). Values presented as median (interquartile range) or n (%). AJC, Active Joint Count; cJADAS10, clinical Juvenile Arthritis Disease Activity Score 10 joints; csDMARD, conventional synthetic Disease‐Modifying Antirheumatic Drug; JIA, Juvenile Idiopathic Arthritis; PhGA, Physician Global Assessment; PPGA, Patient/Parent Global Assessment.

^a^ Statistically significant at *P* < 0.05.

^b^ Data complete for 58%.

^c^ Data complete for 89% (formal JIA subtype yet unknown at first hospital visit).

^d^ Kruskal‐Wallis rank sum test.

^e^ Pearson's chi‐square test.

^f^ Fisher's exact test.

### Effect of time to biologic treatment on reaching inactive arthritis at six months

**Figure 1:**
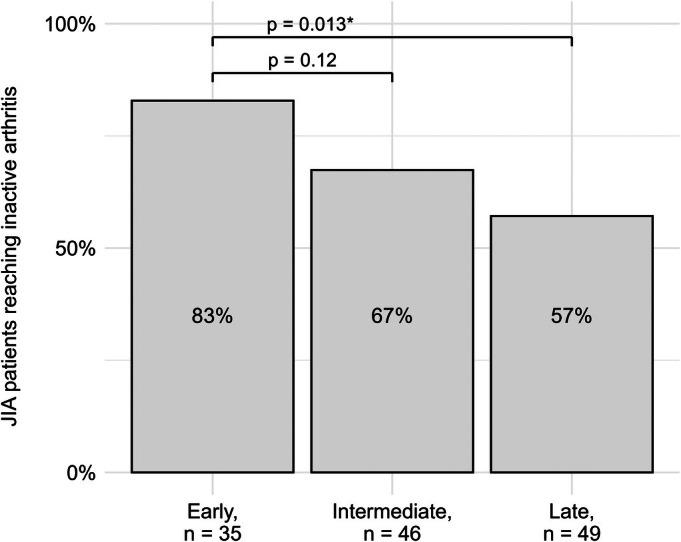
Proportion of patients with JIA reaching inactive arthritis (AJC = 0) after six months of early biologic treatment compared to intermediate and late biologic treatment. Proportion of patients with JIA reaching inactive arthritis after six months of biologic treatment across three treatment groups: early treatment group (time from symptom onset to biologic start: 0–6 months), intermediate treatment group (7–12 months), and late treatment group (13–24 months). Inactive arthritis was defined as having an AJC of zero at six months. The statistical significance of the observed differences was tested with the chi‐square test. *Statistically significant at *P* < 0.05. AJC, Active Joint Count; JIA, Juvenile Idiopathic Arthritis.

The early treatment group received biologic treatment at a median of 4 months (IQR: 2.8–5.1), the intermediate group at 8.6 months (IQR: 7.3–9.5), and the late treatment group at 16.8 months (IQR: 14.7–19.0) (Table 1). The proportion of patients reaching inactive arthritis at six months after start of biologic treatment was the highest in the early treatment group (83%) compared to the intermediate (67%, Pearson's chi‐square test, *P* = 0.12) and late treatment group (57%, Pearson's chi‐square test, *P* = 0.013, Figure 1). Of the 88 patients with inactive arthritis, 4 still had active uveitis (physician reported) after six months of biologic treatment. To account for extra‐articular manifestations, the proportion of patients reaching AJC + PhGA <1 was measured, resulting in comparable results (83% of the patients reaching AJC + PhGA <1 in the early treatment group, 57% in the intermediate treatment group, and 53% in the late treatment group; Supplementary Figure 3).

### Multivariate logistic regression model for active arthritis at six months

The time between symptom onset and the start of biologic treatment had a significant effect on the probability of reaching inactive arthritis at six months after treatment start (Figure 1 and Table 3). To assess the corrected effect of time between symptom onset and biologic start on the probability of reaching inactive arthritis six months after starting biologic treatment, a multivariate logistic regression model was created. Univariate analysis identified lower AJC and PhGA at the start of biologic therapy as significantly associated with reaching inactive arthritis (Wilcoxon rank sum test, *P* < 0.05, Table 3). Both were included in the initial model with an interaction term between these variables to account for nonindependence. Prior treatment with csDMARDs, although not statistically significant in the univariate analysis (Table 3), was also included in the initial multivariate model because of its possible effect on subsequent treatment response. After backward step‐wise selection, the model included AJC interacting with PhGA next to the time between symptom onset and biologic start. Variance inflation factor calculations showed that collinearity was acceptable in the model without the interaction term. Therefore, the final model included AJC and PhGA next to time between symptom onset and biologic start.

**Table 3:** Comparison of patient‐, disease‐, and treatment‐related factors at the start of biologic treatment between patients with inactive and active arthritis six months after biologic treatment in JIA*

	Active arthritis (AJC ≥ 1) n = 42	Inactive arthritis (AJC = 0) n = 88	*P* value
Age at onset	11.4 (4.1, 13.7)	10.3 (3.7, 13.4)	0.3^c^
Sex (female)	28 (67)	58 (66)	>0.9^d^
Country of residence (the Netherlands)	30 (71)	68 (77)	0.5^d^
AJC	6.0 (3.0, 9.0)	3.5 (2.0, 6.0)	0.013^a^ ^,^ ^c^
PhGA	4.00 (3.00, 5.58)	3.00 (2.00, 4.63)	0.018^a^ ^,^ ^c^
PPGA^b^	6.00 (4.00, 7.00)	4.00 (1.69, 6.43)	0.077^c^
Months to biologic start	11.7 (7.3, 16.6)	8.8 (5.2, 13.5)	0.034^a^ ^,^ ^c^
csDMARDs use before biologics	29 (69)	54 (61)	0.4^d^
csDMARDs continued or started	30 (71)	65 (74)	0.8^d^
Biologic therapy			>0.9^e^
TNFa inhibition	41 (98)	85 (97)	
IL‐6 inhibition	1 (2.4)	2 (2.3)	
JAK inhibition	0 (0)	1 (1.1)	
JIA subtype			0.8^e^
Enthesitis arthritis	10 (24)	21 (24)	
Oligoarticular course JIA	13 (31)	30 (34)	
Polyarticular course JIA	16 (38)	34 (39)	
Psoriatic arthritis	2 (4.8)	1 (1.1)	
Undifferentiated JIA	1 (2.4)	2 (2.3)	

* Patient characteristics were measured at the start of biologic treatment. Values presented as median (interquartile range) and n (%). AJC, Active Joint Count; csDMARD, conventional synthetic Disease‐Modifying Antirheumatic Drug; IL‐6, Interleukin‐6; JIA, Juvenile Idiopathic Arthritis; PhGA, Physician Global Assessment; PPGA, Patient/Parent Global Assessment; TNFα, Tumor Necrosis Factor α.

^a^ Statistically significant at *P* < 0.05.

^b^ Data complete for 68% of the patients.

^c^ Wilcoxon rank sum test.

^d^ Pearson's chi‐square test.

^e^ Fisher's exact test.

Although PPGA at the start of biologic treatment was not significant in the univariate analysis, PPGA at the start of biologic treatment tended to be lower for children reaching inactive arthritis. For this reason, a separate model was tested with PPGA as an extra variable next to AJC, PhGA, and time to biologic treatment. In this analysis, only those patients for whom PPGA was available were included (89 of 130 patients, 29 with active and 60 with inactive arthritis, corresponding to 69% and 68% of all patients with inactive and active arthritis, respectively). The inclusion of PPGA next to AJC, PhGA, and time to biologic treatment did show similar results compared to the model without PPGA.

### Visualization of risk model

**Figure 2:**
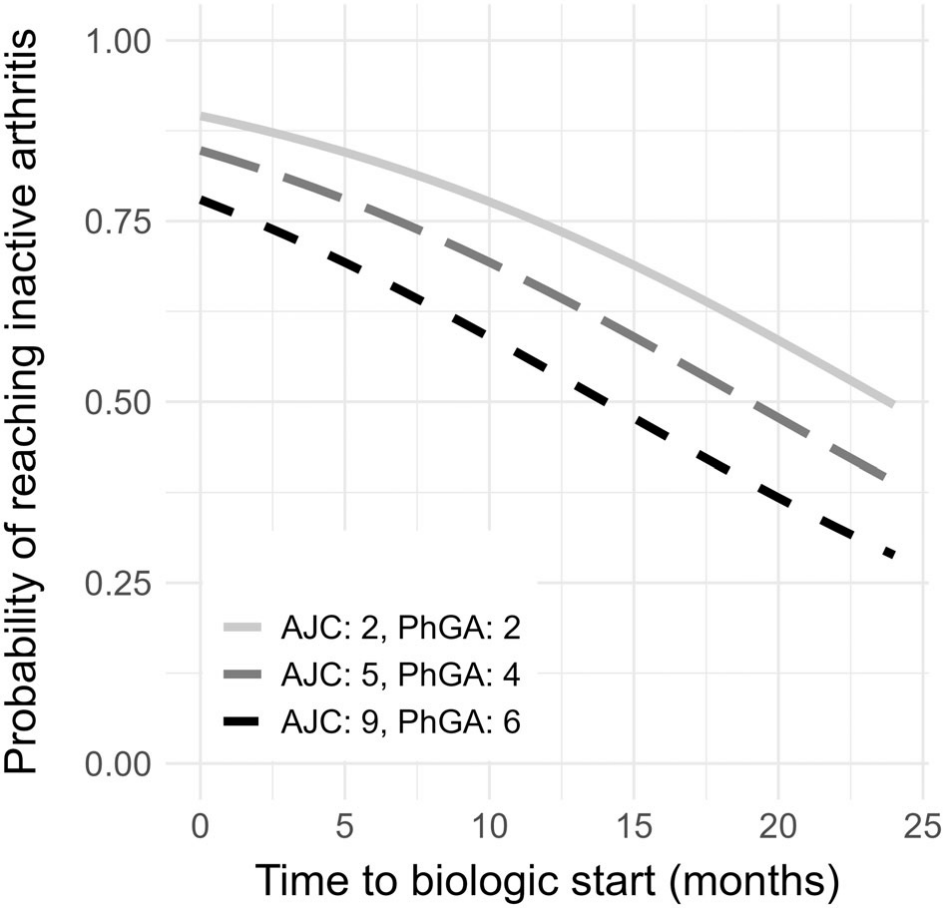
Visualization of the impact of AJC, PhGA at the start of biologic therapy, and time from symptom onset to biologic start on the probability of reaching inactive arthritis (AJC = 0) after six months of biologic treatment for patients with JIA. Effect of time to biologic start on the probability of reaching inactive arthritis in patients with JIA after six months of biologic therapy. Values for AJC and PhGA at the start of biologic therapy were based on the more extreme phenotypes within the interquartile range of both groups. Values for AJC, PhGA, and time were entered into the described model for visualization of the effect of these variables on the probability of response. The calculated probability of response is depicted. AJC, Active Joint Count; JIA, Juvenile Idiopathic Arthritis; PhGA, Physician Global Assessment.

The model shows that active arthritis at six months was associated with time between symptom onset and biologic start after adjusting for AJC and PhGA (adjusted odds ratio per month: 1.09 [confidence interval 1.02–1.17]; Table 4). Odds ratios for AJC and PhGA at the start of treatment are shown in Table 4. Similarly, in a separate model using AJC and PhGA <1 as the outcome, comparable associations with time between symptom onset and biologic start were observed after adjusting for AJC and PhGA (Supplementary Table 2). To visualize the effect of time between symptom onset and biologic start adjusted for AJC and PhGA, a graphical representation is shown in Figure 2. The graphical representation of the model shows how the chance of reaching inactive arthritis at six months decreases with time for patients with more extreme values for AJC and PhGA at the start of biologic treatment (Figure 2).

**Table 4:** Coefficients table of the multivariate logistic regression model to estimate the effect of time to biologic start in months on having active arthritis at six months of treatment in Juvenile Idiopathic Arthritis corrected for active joint count and the physician global assessment*

Variables at the start of biologic therapy	Crude/adjusted	Estimate	SE	*p*‐value	OR	CI (95%)
Time to biologic start (per month)	Crude	0.069	0.032	0.032	1.07	1.01–1.14
Time to biologic start per month)	Adjusted	0.090	0.035	0.0089	1.09	1.02–1.17
AJC	Adjusted	0.021	0.027	0.438	1.02	0.97–1.08
PhGA	Adjusted	0.185	0.109	0.089	1.20	0.97–1.49

* Odds ratio for failing to reach clinical inactive arthritis at six months after the start of biologic treatment.

Abbreviations: AJC, Active Joint Count; CI, Confidence Interval; OR, Odds Ratio; PhGA, Physician Global Assessment; SE, Standard Error.

## DISCUSSION

This study provides real‐life evidence that the delay in biologic treatment negatively impacts the treatment response in children with nonsystemic JIA. The percentage of children with inactive arthritis at six months after the start of biologics decreased dramatically from 83% to 57% comparing early versus late biologic treatment starters. Moreover, the regression model revealed that for each month of delay to the start of biologic treatment, the odds for still having active arthritis after six months of treatment increases by factor 1.09. These results suggest a window of opportunity to control nonsystemic JIA in which the biologic therapy is initiated early after symptom onset.

Biologic therapies are highly effective in JIA. This study demonstrated that even in real‐life settings, inactive arthritis can be achieved in more than 80% of children across all nonsystemic disease subtypes. These findings mirror findings in patients with sJIA, in whom early start of the interleukin‐1 inhibitor anakinra resulted in rapid and sustained control of disease activity.^18^ In contrast, delayed starters of biologics in sJIA had an eight‐fold higher risk of being a nonresponder.^18^ Achieving these results required a paradigm change—a switch from step‐up therapy approaches from conventional nonsteroidal anti‐inflammatory drugs to csDMARDs to finally biologics for those with polyarticular disease course of sJIA, commonly also viewed as trial‐and‐error strategies—to the early use of biologic therapies for patients with sJIA, the most severe subtype of JIA.

The potential existence of a window of opportunity in patients with rheumatic arthritis has been described previously.^13^, ^19^, ^20^ According to this theory, the disease becomes more difficult to treat when the disease duration is longer because the disease progresses irreversibly. In adults with rheumatic arthritis, early adalimumab start was more successful than the treatment of patients with long disease duration.^21^ In a randomized controlled trial in 85 patients with polyarticular JIA, the chance of reaching clinically inactive disease at six months increased by 32% for each month earlier that aggressive treatment (MTX with etanercept and prednisolone) was started after disease onset.^22^ A three‐arm, single‐blinded, treat‐to‐target study in patients with nonsystemic JIA did not find different remission rates between the treatment groups that started with etanercept directly after diagnosis compared to the two groups starting on csDMARD with or without prednisolone. However, the time from symptom onset to biologic was not investigated. Moreover, this study showed that the majority of patients in all three groups ended up with biologic treatment during the 24‐month study because the goal of inactive disease was not reached without it.^23^ In addition, patients with nonsystemic JIA with an early biologic treatment are more likely to be in drug‐free remission in adulthood compared to patients with a late biologic treatment start (more than five years).^24^, ^25^

Our findings support the theory of a window of opportunity in patients with nonsystemic JIA as well, showing that for patients who will start a biologic, an early start of the therapy is beneficial for the patient's treatment response at least in the first six months after the start of biologic treatment. The study provides evidence that the treatment of patients with nonsystemic JIA may need a similar paradigm shift.

There are several limitations to the study. Our study (with prospectively collected data) cannot rule out that the more severe cases of JIA are overrepresented in the early biologic treatment group. It is not unlikely that at the first outpatient clinic visit of a patient with severe JIA, a physician decides to ignore csDMARDs and start a biologic immediately, whereas less severe patients will start on csDMARDs and will only receive a biologic after several months. To test whether the patients differed at the moment of first therapy choice, a subanalysis was conducted on patients from whom a treatment‐naive (both csDMARDs and biologics) diagnosis visit was available. This analysis showed that the early, intermediate, and late treatment groups were comparable in disease activity (AJC, PhGA, PPGA, and cJADAS10), JIA subtype, age, and biologic sex when the first treatment was initiated at the hospital. This suggests that patients in all three groups had a comparable probability for early start of biologic treatment.

Secondly, we did correct for prior csDMARD use in our analysis, but we did not consider the exact duration of csDMARD therapy or the time between csDMARD therapy start and escalation to biologic treatment. According to the theory regarding the window of opportunity, early treatment with any anti‐inflammatory agent is important to prevent further disease progress. More research is needed to further study the effect of prior csDMARD use on the efficacy of biologic therapy in patients with nonsystemic JIA.

In conclusion, this study shows that the time from symptom onset to biologic treatment negatively affects the treatment response in patients with nonsystemic JIA. These findings support the theory of a window of opportunity for successful timing of biologic treatment in patients with active JIA. The treatment of patients with JIA will be improved when patients who are in need of biologics could start early with biologic treatment without losing time on other treatment strategies first.

## AUTHOR CONTRIBUTIONS

All authors contributed to at least one of the following manuscript preparation roles: conceptualization AND/OR methodology, software, investigation, formal analysis, data curation, visualization, and validation AND drafting or reviewing/editing the final draft. As corresponding author, de Jonge confirms that all authors have provided the final approval of the version to be published and takes responsibility for the affirmations regarding article submission (eg, not under consideration by another journal), the integrity of the data presented, and the statements regarding compliance with institutional review board/Declaration of Helsinki requirements.

## Supporting information


**Disclosure Form**:

**Supplementary Figure 1** Graphical representation of the timing between symptom onset, biologic start, and moment of outcome measurement in the three treatment groups.

**Supplementary Figure 2** Scatterplot of the observed and predicted time between symptom onset and the start of biologic treatment in Juvenile Idiopathic Arthritis patients.

**Supplementary Figure 3** Proportion of Juvenile Idiopathic Arthritis patients reaching an active joint count and physician global assessment score <1 after six months of early biologic treatment compared to intermediate and late biologic treatment.

**Supplementary Table 1** Comparison of clinical characteristics between Juvenile Idiopathic Arthritis patients included and excluded from analysis because the follow‐up visit was outside of the defined timeframe (4 to 8 months after biologic treatment start).

**Supplementary Table 2** Coefficients table of the multivariate logistic regression model to estimate the effect of time to biologic start in months on having active joint count + physician global assessment score ≥ 1 at six months of treatment in Juvenile Idiopathic Arthritis corrected for active joint count and the physician global assessment.

## APPENDIX A MEMBERS OF THE UCAN CAN‐DU AND UCAN CURE CONSORTIA

Members of the UCAN CAN‐DU and UCAN CURE consortia are as follows: Adam Huber, Bianca Lang, Chelsea DeCoste, Elizabeth Stringer, and Suzanne Ramsey (IWK Health Centre); Alan Rosenberg, Kate Neufeld, Mehul Jariwala, and Tristan Kerr (University of Saskatchewan); Alexander Mosoiu, Alisa Rachlis, Amy Xu, Arthur Cheng, Brenleigh Jebb, Brian Feldman, Bruno Pereira, Deborah Levy, Dilan Dissanayake, Elizaveta Limenis, Evelyn Rozenblyum, Harper Cheng, Jennifer Ji Young Lee, Lynn Spiegel, Rayfel Schneider, Ronald Laxer, Ruud Verstegen, Shirley Tse, and Trang Duong (The Hospital for Sick Children); Andrea Human, David Cabral, Herman Tam, Jaime Guzman, Kim Morishita, Kristin Houghton, Lori Tucker, Mercedes Chan, Ross Petty, and Tommy Gerschman (British Columbia Children's Hospital); Annet van Royen‐Kerkhof, Berent Prakken, Erika Van Nieuwenhove, Marc Jansen, and Nico Wulffraat (Wilhelmina Children's Hospital, University Medical Center Utrecht); Ciarán Duffy, Nadia Luca, Roman Jurencak, and Tala El Tal (Children's Hospital of Eastern Ontario); Claire LeBlanc, Gaëlle Chédeville, Piya Lahiry, Rosie Scuccimarri, and Sarah Campillo (Montreal Children's Hospital); Clare Hutchinson (North York General Hospital); Daniah Basodan, Dax G. Rumsey, Hon Yan Ng, Jeanine McColl, Lillian Lim, and Tara McGrath (Stollery Children's Hospital); Danielle Brinkman and Petra Hissink Muller (Willem‐Alexander Children's Hospital, Leiden University Medical Center); Elizabeth Legger and Wineke Armbrust (Beatrix Children's Hospital, University Medical Center Groningen); Ellen Schatorje and Esther Hoppenreijs (Amalia Children's Hospital, Radboudumc and Sint Maartenskinderkliniek); Elodie Boudes, Gillian Currie, Heinrike Schmeling, Muhammed Dhalla, Nicole Johnson, Paivi Miettunen, Ravneet Sran, and Rebeka Stevenson (University of Calgary); Erkan Demirkaya, Jonathan Park, and Roberta Berard (Children's Hospital, London Health Sciences Centre); Giske Biesbroek and Mariken Gruppen (Emma Children's Hospital, Amsterdam University Medical Centers); Gordon Soon (North York General Hospital/Health Sciences North); Joseph Cafazzo (Centre for Global eHealth Innovation, University Health Network, University of Toronto); Liane Heale, Michelle Batthish, and Tania Cellucci (McMaster Children's Hospital); Lily Lim (Children's Hospital Health Centre Winnipeg); Maarten IJzerman (Erasmus University and University of Melbourne); Marinka Twilt (University of Calgary and Alberta Children's Hospital); Marleen Verkaaik, Philomine van Pelt, and Sylvia Kamphuis (Sophia Children's Hospital, Erasmus University Medical Center); Michelle Kip (University of Twente); Nickolas Blanchette (Trillium Health Partners); Paul Dancey (Janeway Children's/Memorial University); and Regina de Geus (University Medical Center Utrecht).
