# Supplementary material for: Effect of Time to Start of Biologic Therapy on Treatment Response in Childhood Arthritis: Results From the UCAN CAN‐DU Cohort
Source: Arthritis Rheumatol. 2026 Jan 16;78(3):743–51. doi: 10.1002/art.43401 (PMC12991923; doi:10.1002/art.43401)
Supplement: Supplementary file 2 — Supplementary Figure 1 Graphical representation of the timing between symptom onset, biologic start, and moment of outcome measurement in the three treatment groups. [file ART-78-743-s001.pdf]

Supplementary Figure 1

Graphical representation of the timing between symptom onset, biologic start, and moment of outcome measurement in the three treatment groups.

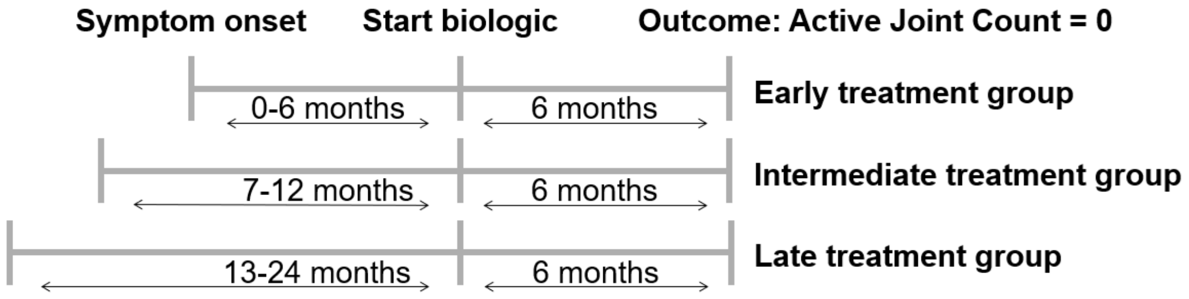

Juvenile Idiopathic Arthritis patients were divided into three different treatment groups. The early, intermediate, and late treatment groups had a time between symptom onset to biologic treatment of 0 to 6 months, 7 to 12 months, and 13 to 24 months, respectively. All patients had a follow-up assessment six months (± 2 months) after biologic start.
