# Supplementary material for: Effect of Time to Start of Biologic Therapy on Treatment Response in Childhood Arthritis: Results From the UCAN CAN‐DU Cohort
Source: Arthritis Rheumatol. 2026 Jan 16;78(3):743–51. doi: 10.1002/art.43401 (PMC12991923; doi:10.1002/art.43401)
Supplement: Supplementary file 3 — Supplementary Figure 2 Scatterplot of the observed and predicted time between symptom onset and the start of biologic treatment in Juvenile Idiopathic Arthritis patients. [file ART-78-743-s002.pdf]

Supplementary Figure 2

Scatterplot of the observed and predicted time between symptom onset and the start of biologic treatment in Juvenile Idiopathic Arthritis patients.

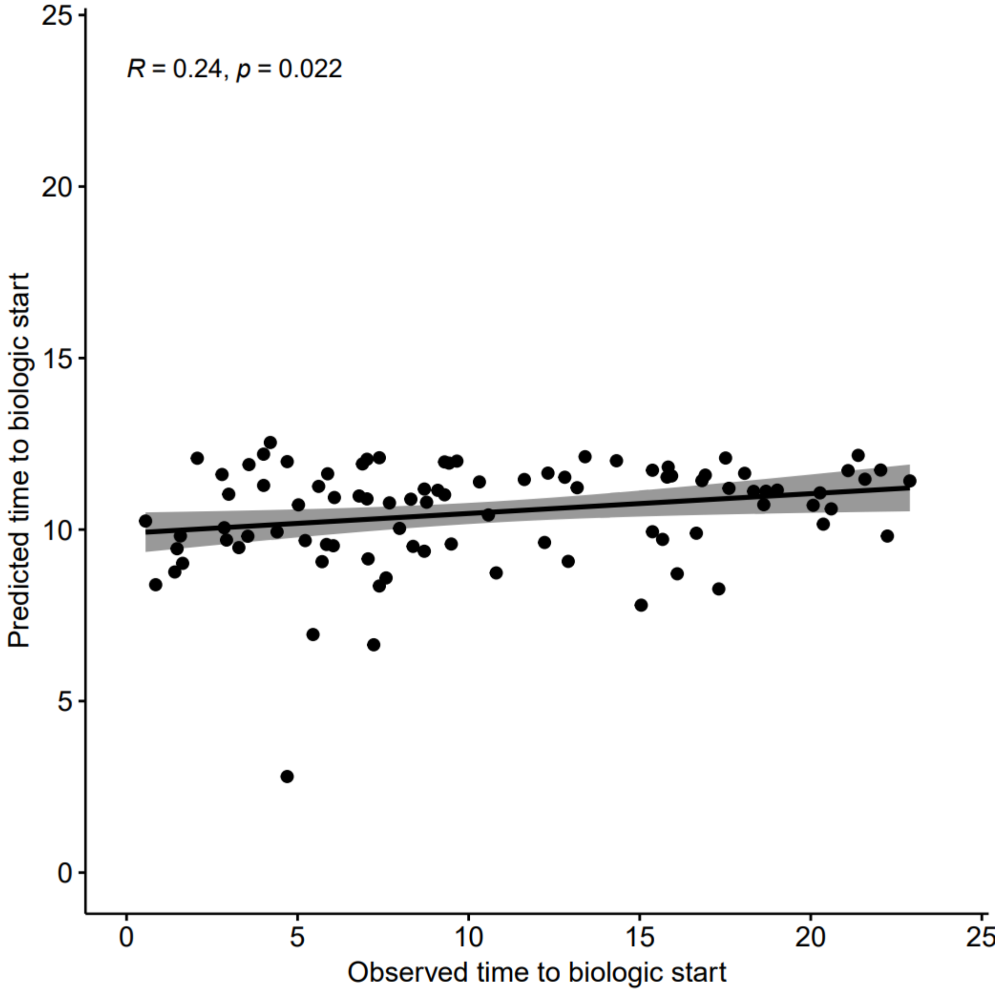

Scatter plot of the observed and predicted time between symptom onset and the start of biologic treatment in Juvenile Idiopathic Arthritis patients. Predicted months to biologic start is analysed with a multivariate linear model including the variables 'Age at symptom onset', 'Active Joint Count', and 'Physician global assessment' measured at the first hospital visit of treatment-naive JIA patients. Weak correlation was found between the observed and predicted time between symptom onset and the start of biologic treatment (R = 0.24).
