# Supplementary material for: Effect of Time to Start of Biologic Therapy on Treatment Response in Childhood Arthritis: Results From the UCAN CAN‐DU Cohort
Source: Arthritis Rheumatol. 2026 Jan 16;78(3):743–51. doi: 10.1002/art.43401 (PMC12991923; doi:10.1002/art.43401)
Supplement: Supplementary file 4 — Supplementary Figure 3 Proportion of Juvenile Idiopathic Arthritis patients reaching an active joint count and physician global assessment score <1 after six months of early biologic treatment compared to intermediate and late biologic treatment. [file ART-78-743-s005.pdf]

Supplementary Figure 3

Proportion of Juvenile Idiopathic Arthritis patients reaching an active joint count and physician global assessment score <1 after six months of early biologic treatment compared to intermediate and late biologic treatment.

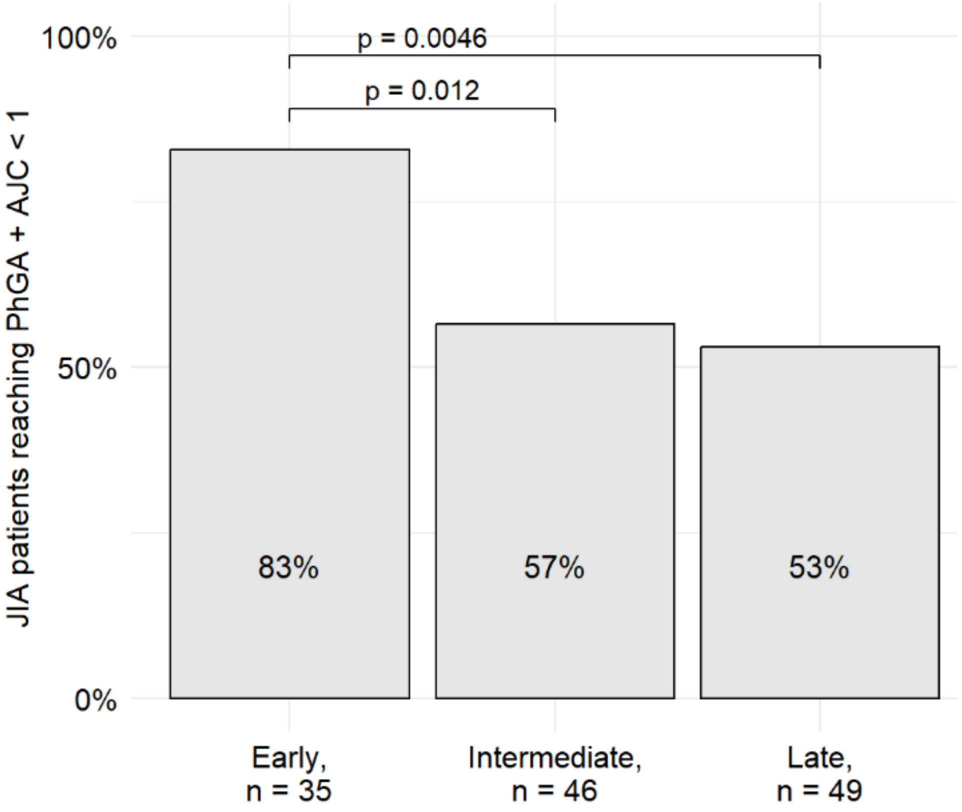

Proportion of JIA patients reaching an active joint count and physician global assessment score <1 after six months of biologic treatment across three treatment groups: early treatment group (time from symptom onset to biologic start: 0-6 months), intermediate treatment group (7-12 months), and late treatment group (13-24 months). The statistical significance of the observed differences was tested with the Chi-Squared test.
