# Supplementary material for: Effect of Time to Start of Biologic Therapy on Treatment Response in Childhood Arthritis: Results From the UCAN CAN‐DU Cohort
Source: Arthritis Rheumatol. 2026 Jan 16;78(3):743–51. doi: 10.1002/art.43401 (PMC12991923; doi:10.1002/art.43401)
Supplement: Supplementary file 5 — Supplementary Table 1 Comparison of clinical characteristics between Juvenile Idiopathic Arthritis patients included and excluded from analysis because the follow‐up visit was outside of the defined timeframe (4 to 8 months after biologic treatment start). [file ART-78-743-s004.pdf]

Supplementary Table 1

Comparison of clinical characteristics between Juvenile Idiopathic Arthritis patients included and excluded from analysis because the follow-up visit was outside of the defined timeframe (4 to 8 months after biologic treatment start).

**Characteristic**	**N**	**Overall**, N = 155	**Excluded**, N = 25	**Included**, N = 130	**p-value**
Active Joint Count	155	4.0 (2.0, 7.0)	4.0 (2.0, 7.0)	4.0 (2.0, 7.0)	0.7
Physician global assessment	155	3.00 (2.00, 5.00)	3.00 (2.00, 4.00)	3.35 (2.00, 5.00)	0.3
Patient/Parent global assessment	109	4.20 (2.00, 6.50)	3.75 (1.68, 5.00)	4.80 (2.40, 6.80)	0.12
cJADAS10	109	12.0 (8.3, 16.5)	10.9 (7.5, 12.9)	12.5 (8.9, 17.0)	0.10

Legend: Median (Interquartile range), Wilcoxon rank sum test. cJADAS: clinical Juvenile Arthritis Disease Activity Score.
