# Supplementary material for: Effect of Time to Start of Biologic Therapy on Treatment Response in Childhood Arthritis: Results From the UCAN CAN‐DU Cohort
Source: Arthritis Rheumatol. 2026 Jan 16;78(3):743–51. doi: 10.1002/art.43401 (PMC12991923; doi:10.1002/art.43401)
Supplement: Supplementary file 6 — Supplementary Table 2 Coefficients table of the multivariate logistic regression model to estimate the effect of time to biologic start in months on having active joint count + physician global assessment score ≥ 1 at six months of treatment in Juvenile Idiopathic Arthritis corrected for active joint count and the physician global assessment. [file ART-78-743-s003.pdf]

**Supplementary Table 2**

**Coefficients table of the multivariate logistic regression model to estimate the effect of time to biologic start in months on having active joint count + physician global assessment score ≥ 1 at six months of treatment in JIA corrected for AJC and PhGA.**

| Variables at start of biologic therapy | Crude/ Adjusted | Estimate | SE | p-value | OR | CI (95%) |
|---|---|---|---|---|---|---|
| Time to biologic start (per month) | Crude | 0.071 | 0.032 | 0.024 | 1.07 | 1.01-1.14 |
| Time to biologic start (per month) | Adjusted | 0.096 | 0.034 | 0.0049 | 1.10 | 1.03-1.18 |
| AJC | Adjusted | 0.017 | 0.027 | 0.527 | 1.02 | 0.97-1.08 |
| PhGA | Adjusted | 0.231 | 0.107 | 0.031 | 1.26 | 1.03-1.57 |

Legend: Odds ratio for failing to reach an active joint count + physician global assessment score <1 at six months after the start of biologic treatment. Abbreviations: AJC: Active Joint Count. JIA: Juvenile Idiopathic Arthritis. PhGA: Physician Global Assessment. SE: Standard Error. OR: Odds Ratio. CI: Confidence Interval.
